# Complementarity in mixed farming systems enhances the smallholders income: Evidence from Punjab, Pakistan

**DOI:** 10.1371/journal.pone.0319995

**Published:** 2025-04-04

**Authors:** M’hand Fares, Saqlain Raza, Tusawar Iftikhar Ahmad

**Affiliations:** 1 INRAE-ACT, UMR SELMET, Montpellier, France; 2 Imam Abdulrahman bin Faisal University, College of Applied Medical Science Jubail, Dammam, Kingdom of Saudi Arabia; 3 The Islamia University of Bahawalpur, Bahawalpur, Punjab, Pakistan; Federal University of Agriculture Abeokuta, NIGERIA

## Abstract

Mixed cropping and livestock production is a widespread farming system in less developed countries. The literature has mainly highlighted the synergistic effects between crop and livestock systems from an agronomic and environmental point of view, but has never investigated the (economic) complementarity that may exist between the two activities. Complementarity exists when mixed farming allows smallholders to earn higher incomes than in specialized systems, i.e., crop-only or livestock-only. Our paper is the first to test for complementarity in mixed farming by deriving empirical predictions from the theory of supermodularity, which are tested econometrically using a database of 360 farming households in the Punjab province of Pakistan. Our estimation results confirm the existence of a significant and positive complementary effect between crop and livestock activities, and also provide a direct measure of this effect. The smallholder can earn an average additional income of 791 rupees (out of an average total income of 12,010 rupees) by choosing mixed farming. This implies that smallholders adopt mixed farming not only for its agronomic and environmental benefits, but also because it can generate higher incomes than specialized farming systems to alleviate smallholder poverty. Apart from the choice of activity, our estimation results show that the other variables that significantly increase smallholder incomes are the education level of the household head, as well as access to urban markets, herd size, and land size. We also find that the positive impact of land expansion does not depend on the property rights regime, i.e., the additional land can be owned or rented (sharecropping). A specific public policy aimed at reducing smallholder poverty must prioritize the improvement of these key factors, especially access to urban markets and sharecropping.

## Introduction

For at least two decades, research and development strategies have explored the importance of mixed farming systems in providing crop and livestock products and their potential to address environmental challenges, food security and poverty alleviation in developing countries [1–3]. This is especially true because two-thirds of the world’s rural poor depend on mixed farming for their livelihoods [1,4–7]. Integrating crop and livestock systems is also one of several agricultural strategies to restore agricultural diversity and improve ecosystem services [8,9].

Integrated farming systems allow intensification of production and income, reduce adverse environment impact and benefit poor farmers [7,10–13]. Mixed crop-livestock systems are favored due to resource availability, nutrient recycling among farm components, and reduced variability in cash flows [14–16]. The synergistic role of different components of mixed crop-livestock systems on smallholdings has been reported [17], e.g., crop-livestock integration can allow diversification of production and better distribution of labor throughout the year, as well as distribution of tasks among different components of the household [7]. Thus, integrated crop-livestock systems are regaining interest worldwide due to their economic and environmental benefits [15,18,19].

However, a common view is that crop/livestock productivity (such as crop or milk production per animal per day, growth and reproduction rates) is lower in mixed systems than in specialized systems. This has sometimes led to the interpretation that mixed systems are less productive. However, although there may be lower productivity per unit of land or animal on a farm, overall higher productivity is common due to synergy between tasks, products, and inputs or reduction of inputs [20].

According to [21], the synergistic interactions of the components of the mixed farming system may also have a significant and positive economic impact. The fact that smallholders adopt crop and livestock simultaneously may indicate that these activities are complementary, i.e., the marginal returns to one activity increase as the intensity of the other increases. Although several empirical studies have examined the integration of crop and livestock activities [22,23], there is no paper that tests for this possible complementary effect.

Our paper provides an original contribution by testing the complementarity effect between crop and livestock activities in the Punjab province of Pakistan. The theoretical framework of complementarity is based on the theory of supermodularity [24]. It shows that when a function is supermodular, it exhibits a complementarity effect. That is, the payoff of joint strategies is greater than the sum of the payoffs of those strategies taken separately.

To test for this complementary effect in the mixed cropping and livestock system, [25] define two empirical approaches derived from a unified supermodularity framework: the adoption (indirect) approach and the production (direct) approach. The adoption approach is mainly used by default when no performance measure is available. In contrast, the production approach, which we use in this paper, is concerned when a performance measure in terms of income is available. In this case, we use ordinary least squares (OLS) estimation and regress income on exogenous variables and dummies representing the different strategies [26,27]. A complementarity test is performed on the coefficient associated with these dummies to see if the marginal return of one activity increases when the other activity is adopted.

To estimate the linear regression model with OLS method, we use data collected from three regions in Punjab, Pakistan. Punjab is the largest Pakistan province in terms of its share in agriculture (crop and livestock production). Its rural population is mainly composed of smallholder farmers who own less agricultural land and use different farming systems: crop-only, livestock-only, or mixed farming systems. The survey data collected on this population includes information on the farming activity chosen by the smallholder, as well as variables on households, land and livestock resources, and the region or zone of production. The database used in this study includes 360 observations of smallholder households.

Our paper makes a substantial contribution to the study of mixed farming by implementing a pioneering approach, complementarity, to assess the income gains for smallholder farmers resulting from the integration of crop and livestock activities within farming systems. Furthermore, the complementarity approach allows us to integrate the findings from the previous literature on risk mitigation and buffer mechanisms against losses due to the integration of livestock in crop farming systems [14,28–33]. This suggests that, in addition to the income generated from integrating different activities, the livestock activity provides farmers with insurance against climatic and price risks, which can improve their well-being.

The remaining sections are organized as follows. Section 2 reviews the related literature on economic explanations of crop-livestock integration and mixed farming system efficiency. Section 3 presents the study area, the sampling procedure and the data collection method. The theory of complementarity and the empirical approach for testing complementarity are presented in Section 4. The econometric results and their discussion are presented in section 5. Finally, section 6 provides some concluding remarks.

### Related literature

Most studies of rural household market participation focus on either crop or livestock markets separately [34,35]. Thus, in explaining smallholder participation in crop markets, livestock is usually included in the crop market participation equation as a draught power, a risk insurance indicator, or as an alternative source of income to crop sales.

Some papers focus on the efficiency of mixed farming systems where both activities are jointly adopted [12]. For example, the mixed system can better manage risk through diversification and consumption smoothing. Mixed cropping and livestock production can help farmers become more resilient to climate change, as mixing crops and livestock often leads to more efficient use of natural resources [36,37]. Livestock can also provide a buffer against losses in a given season. For example, selling a few sheep or goats can help a family cope with a poor grain harvest [28]. Australian farmers are motivated by the risk mitigation benefits of mixed farming to reduce income volatility due to both price and climate variability [14]. Integrated farming systems can be less risky because of synergies between enterprises, diversity of products, and environmental soundness [38].

More generally, since climate risk management tools are imperfect or absent in developing countries, the appropriate measures to control the distribution of risk variables are farm diversification [29] and risk-controlling inputs [30]. [31] has shown the importance of livestock as a consumption smoothing measure for income and price risks. He also found that the increase in the livestock subsector’s share of agricultural value added should have improved the welfare position of poorer households in rural areas. [32] also highlights the role of livestock in agriculture as a risk management tool and evidence of a rural insurance mechanism through income diversification [33]. For example, income from milk and dairy products has been estimated at 27% of household income, and the sale of animals during the religious festival of Eid-ul-Azha is considered one of the best returns from livestock [39].

In this paper, we depart from the previous literature that explains the adoption of mixed farming in terms of its insurance mechanism against climatic or price risks. Rather, our hypothesis is that smallholders may adopt mixed farming because crop and livestock activities are complementary, and thus the payoff from joint adoption of these activities is higher than the sum of the payoffs from each activity separately. To test for this complementary effect, we use the complementary framework derived from supermodularity theory.

## Materials and methods

### Study area and sampling procedure

The data for this study come from a household survey conducted by the Ministry of Planning and Development (P&D), Government of Punjab, Pakistan. The survey was conducted in 2010 in the province of Punjab, Pakistan.

Out of the four provinces, Punjab is the second largest province of Pakistan. It contributes more than 50 percent of Pakistan’s GDP and is home to 56 percent of its total population. The administrative structure of Punjab constitutes 36 districts further divided into 164 *tehsils*, an administrative subdivision of a district. The number of villages in every *tehsil* depends on its population density and geographical area. The province is also divided into three agro-climatic zones on the basis of differences in cropping pattern, irrigation facilities, soil type, groundwater table and rainfall pattern in different parts of the province. These zones are named as mixed cropping Punjab, rainfed (barani) Punjab, and cotton/wheat Punjab [40].

In order to select households (as units of survey), a four-stage random stratified sampling technique was applied. In the first stage, 3 out of the 36 districts were selected from the entire province to represent the three agro-climatic zones. In order to control for social and economic disparities that occur across the province within and amongst various districts and *tehsils*, and in order to ensure that the selected districts represent maximum and diverse geographical regions of the entire province, the selection of districts was done systematically as opposed to being done randomly. Starting from the North of the province, districts were selected towards the East, West and South of the province. The three selected districts were: Faisalabad from mixed-cropping zone, Chakwal form rain-fed zone and Bahawalnagar from cotton-wheat zone.

In the second stage, four *tehsils* were randomly selected from each identified district: Faisalabad from mixed-cropping zone (*tehsils* 8, 35, 6, 24), Chakwal form rain-fed zone (*tehsils* 1, 21, 5, 30) and Bahawalnagar from cotton-wheat zone (*tehsils* 2, 3, 27, 26). In the third stage, at least two villages were subsequently selected randomly from amongst the selected tehsils; and in the fourth and final stage, participating and non-participating households were selected at random for conducting surveys. A total of 360 households were interviewed. The data collection process involved field visits to the selected villages and completion of the questionnaire by interviewing the target households in face to face. The village survey methodology used in this study was promoted three decades ago as an empirically based alternative to traditional economic analyses of rural situations [41,42]. The data collected was qualitative and quantitative and covered the socio-economic characteristics of the 360 farmers as well as the inputs (land, capital, and labour) and integration decision of crop and livestock activities in the farm.

### Analysis Methods

#### Complementarity between farming activities.

Since we want to investigate the complementary effect that may exist between crop and livestock activities in the Punjab Pakistan farming, we need to define a production framework with possible complementarity between activities.

The complementarity approach traces back to a mathematical theory of supermodularity functions [24], from which we can derive predictions about the complementarity of farmers’ strategies. Suppose that a smallholder farmer makes $S=\left({\text{s}}^{1},{\text{s}}^{2}\right)$ feasible crop and livestock activity choices, where S⊂X and *X* is a partially ordered set, to maximize his income function


$$\begin{array}{c} max_{(s^{1},s^{2})\in S\subset X}F\left(s^{1},s^{2}\right) \end{array}$$ (1)

where $s^{1}$ (crop activity) and $s^{2}$ (livestock activity) are discrete choices with $s^{j}\in \left\{0,1\right\}$, j=1,2, and $s^{j}=1$ indicating the adoption of the activity *j*, $s^{j}=0$ its non-adoption.

A function is said to be supermodular when the change resulting from increasing all the arguments together is higher than the sum of the changes when arguments are increased separately [24]. Therefore, the function F(S) is supermodular, and $s^{1}$ and $s^{2}$ are complementary activities only if


$$\begin{array}{c} F\left(1,1\right)+F\left(0,0\right)\geq F\left(1,0\right)+F\left(0,1\right) \end{array}$$ (2)

That is, adding an activity while the other activity is already being performed has a greater incremental effect on income than adding the activity in isolation. This condition can be rewritten as follows


$$\begin{array}{c} \left[F\left(1,1\right)+F\left(0,0\right)\right]-\left[F\left(1,0\right)+F\left(0,1\right)\right]\geq 0 \end{array}$$ (3)

### Testing for complementarity

Two approaches are commonly used in the literature to test for complementarity [25]. The production approach is based on testing the effect of different combinations of practices, along with observable characteristics, directly on the performance measure. If a performance measure is not available, an adoption approach based on the analysis of the correlation between different activity decisions conditional on a common set of exogenous variables is used [43,44].

Since a performance measure is available in our database, i.e., the household total income from farm or off-farm activities, we use the production approach to test for complementarity. Therefore, the test of complementarity is performed by regressing a measure of performance on a set of dummy variables representing the adoption of different combinations of activities, along with other explanatory variables. The results of the regression provide evidence of complementarity (substitutability) when a significant and positive (negative) coefficient of the joint activities dummy variable is observed. Applying this approach to organizational innovation, [27] directly estimate the objective function and investigate whether research and development make-buy decisions are complementary. Similarly, [26] investigate the complementarity of product, process and organizational innovations and their impact on labor productivity, and [45] suggests a general framework of complementarity between multiple practices in the production approach.

In our context, a smallholder chooses between different combinations of crop and livestock activities $\left(s^{1},s^{2}\right)$. More formally, a smallholder chooses: (i) either crop only $\left(s^{1}=1,s^{2}=0\right)$ or livestock only $\left(s^{1}=0,s^{2}=1\right)$, (ii) both activities together $\left(s^{1}=s^{2}=1\right)$; or (iii) off-farm activity, i.e., neither crop nor livestock activity $\left(s^{1}=s^{2}=0\right)$.

Suppose now that the smallholder production function depends on these combinations of livelihood activities and the “classic” production factors in agriculture, i.e., capital, labor and land. A log Cobb-Douglas specification of such a production function can be written as follows:


$$\begin{array}{c} lnF_{i}\left(K,L,T;s^{1},s^{2}\right)=\alpha {\text{K}}_{\text{i}}+\beta L_{i}+\gamma T_{i}+\left(1-s_{i}^{1}\right)\left(1-s_{i}^{2}\right)\theta_{00}\\ +s_{i}^{1}\left(1-s_{i}^{2}\right)\theta_{10}+\left(1-s_{i}^{1}\right)s_{i}^{2}\theta_{01}+s_{i}^{1}s_{i}^{2}\theta_{11} \end{array}$$ (4)

Where K,L,T represent the capital, labor and land factors, while α,β,γ denote their respective productivity parameters. Regarding the livelihood activity strategies, we have $\left(1-s_{i}^{1}\right)\left(1-s_{i}^{2}\right)\theta_{00}$ when the smallholder chooses off-farm activity $\left(s^{1}=s^{2}=0\right)$, with $\theta_{00}$ the productivity coefficient for off-farm work. Similarly, we have $s_{i}^{1}\left(1-s_{i}^{2}\right)\theta_{10}$ when only livestock activity is chosen $\left(s^{1}=1;s^{2}=0\right)$ with its productivity coefficient $\theta_{10}$ and $\left(1-s_{i}^{2}\right)\theta_{01}$ when crop only activity is chosen $\left(s^{1}=0;s^{2}=1\right)$ with its productivity coefficient $\theta_{01}$. Finally, we have $s_{i}^{1}s_{i}^{2}\theta_{11}$ when crop and livestock activities are jointly adopted (mixed farming) $\left(s^{1}=1;s^{2}=1\right)$, with its productivity coefficient $\theta_{11}$.

To test econometrically for complementarity between activities, we use a linear regression model of equation (5), where the log of the household total income is regressed on the production factors, the alternative combinations of activities, and other exogenous variables.


$$\begin{array}{c} lnF_{i}\left(K,L,T;s^{1},s^{2}\right)=\alpha {\text{K}}_{\text{i}}+\beta L_{i}+\gamma T_{i}+\left(1-s_{i}^{1}\right)\left(1-s_{i}^{2}\right)\theta_{00}+s_{i}^{1}\left(1-s_{i}^{2}\right)\theta_{10}\\ +\left(1-s_{i}^{1}\right)s_{i}^{2}\theta_{01}+s_{i}^{1}s_{i}^{2}\theta_{11}+Z_{i}\delta_{i}+\epsilon_{i} \end{array}$$ (5)

where $Z_{i}$ is the vector of (other) exogenous variables and $\delta_{i}$ its vector of coefficients, $\epsilon_{i}$ are the error terms (unobserved characteristics) distributed as multivariate normal, identically and independently across the i=1,…,n farmers, with zero mean and covariance matrix $\Sigma =\sigma_{i}>0$.

To examine the partial returns from crop and livestock, we rewrite equation (5) as follows


$$\begin{array}{c} lnF_{i}\left(K,L,T;s^{1},s^{2}\right)=\alpha {\text{K}}_{\text{i}}+\beta L_{i}+\gamma T_{i}+\theta_{0}+s_{i}^{1}\theta^{C}+s_{i}^{2}\theta^{L}+s_{i}^{12}\theta^{CL}+Z_{i}{\text{δ}}_{\text{i}}+\epsilon_{i} \end{array}$$ (6)

Where $\theta^{0}=\theta_{00}$; $\theta^{C}=\theta_{01}-\theta_{00}$; $\theta^{L}=\theta_{10}-\theta_{00}$; $\theta^{CL}=\left[\theta_{11}+\theta_{00}\right]-\left[\theta_{01}+\theta_{10}\right]$. That is, $\theta^{0}$ is the constant, $\theta^{C}$ captures the non-exclusive partial returns to crop activity, $\theta^{L}$ is the non-exclusive partial returns to livestock activity and $\theta^{CL}$ the returns to adopting both activities together. This latter is exactly the complementarity parameter we want to estimate. Indeed, we know from equation (3) that the objective function is supermodular and $s^{1}$ and $s^{2}$ are complementary if $[F_{i}\left(K,L,T;1,1\right)+F_{i}\left(K,L,T;0,0\right)]-[F_{i}\left(K,L,T;0,1\right)+F_{i}\left(K,L,T;1,0\right)]\geq 0$. That is, when


$$\begin{array}{c} \theta^{CL}=\left[\theta_{11}+\theta_{00}\right]-\left[\theta_{01}+\theta_{10}\right]\geq 0 \end{array}$$ (7)

Following [25] and the related literature implementing the production approach of complementarity [26,27,45], we use the ordinary least squares (OLS) method to estimate the coefficients of the linear regression model (6).

In terms of comparative static, this linear regression model between activities mainly examines the impact of the choice of activity and its effect on the total income of the smallholder. More precisely, we try to see if the integration of crop and livestock activities leads to a higher income than each activity separately, taking the income from the off-farm activity as a reference. That is, we are agnostic about the sign of each specialized agricultural activity (crop-only or livestock-only) because it is difficult to find a clear theoretical prediction for household income relative to other activities. The only prediction is the one derived from the theory of complementarity. That is, compared to off-farm activities, mixed farming may generate higher income than crop and livestock activities taken in isolation.

Regarding the impact of inputs on total farm income, the Cobb-Douglas specification of our production function simply says that the marginal impact of the three factors of production (land, capital, labor) is positive. Table 1 below shows the predicted signs of the variables.

**Table 1:** Theoretical impact of the variables.

Inputs and activity variables	Expected sign
Mixed farming	(+)
Capital	(+)
Labor	(+)
Land	(+)

### Ethics approval statement

The INRAE Ethics Committee stated that at the time of the survey, it was not possible for INRAE researchers to submit their projects for ethics approval. Furthermore, the project would not currently require specific ethical approval from the INRAE Ethics Committee, based on the ethical self-assessment.

## Results and discussion

### Explained and explanatory variables

Table 2 describes the explained and explanatory variables used in the linear regression model (6) of the production function with complementarity.

**Table 2:** Descriptive statistics of variables.

Variable	Description	Obs.	Mean	Std.Dev	Min.	Max.	Expected impact
INCOME	Logarithmic value of the household income (in thousands of Pak Rupees)	360	12.01	0.733	9.548	14.73	
Crop	equals to 1 if the household is involved in cropping; 0 otherwise	360	0.586	0.493	0.00	1.00	NK
Livest	equals to 1 if the household is involved in livestock; 0 otherwise	360	0.600	0.490	0.00	1.00	NK
CropLive	equals to 1 if the household is involved in cropping and livestock; 0 otherwise	360	0.533	0.499	0.00	1.00	(+)
Others	equals to 1 if the household is involved in other activities than cropping and/or livestock; 0 otherwise [Ref]	360	0.347	0.477	0.00	1.00	Ref
AGE	Age of the head of the household *1 if 20–29 years; 2 if 30–39 years; 3 if 40–49 years; 4 if 50–59 years; 5 if 60–69 years; 6 if 70* + *years*	360	3.272	1.141	1.00	6.00	(+)
EDU	Education level of the head of the household: *1 = Illiterate; 2 = Primary; 3 = Middle; 4 = Secondary; 5 = Higher secondary; 6 = College*	360	2.516	1.451	1.00	6.00	(+)
FmlySys	equals to 1 if the household has joint family; 0 otherwise	360	0.542	0.499	0.00	1.00	(+)
FmlySize	Number of family members	360	5.366	1.743	1.00	11.00	(+)
LandSize	Land owned by a household (in hectares)	360	2.116	3.199	0.00	25.30	(+)
LandOwn	equals to 1 if the household is the owner; 0 otherwise	360	0.800	0.400	0.00	1.00	(+)
CropShare	equals to 1 if the household is sharecropper; 0 otherwise	360	0.144	0.352	0.00	1.00	(+)
FMachinery	N° of machine accessories used by the household	360	0.311	0.946	0.00	6.00	(+)
HerdSize	Number *n* of animals kept by the household = 1 if n∈ [1;10]; = 2 if n∈ [11;20]; = 3 if n∈ [21-30]; = 4 if n∈ [31-40]; = 5 if n> 40;	360	1.172	0.498	1.00	5.00	(+)
Rainfed	equals to 1 if the household lives in rain-fed zone; 0 otherwise	360	0.333	0.472	0.00	1.00	(+)
Mixcrop	equals to 1 if the household lives in mixed-cropping zone; 0 otherwise	360	0.333	0.472	0.00	1.00	(+)
Cotwheat	equals to 1 if the household lives in cotton-wheat zone; 0 otherwise [Ref]	360	0.333	0.472	0.00	1.00	Ref
Proximity	Distance *d* (in km) from the village to the district capital city =1 *if* d>24; =2 *if* $d\in ]12;24]$; =3 *if* $d\in ]6;12]$; =4 *if* d≤6	360	2.5	1.119	1.00	4.00	(+)

NK (not known): there is no expected sign that can be derived from the theoretical framework; Ref: reference variable.

Income (*INCOME*) is used as the explained (dependent) variable in the regression model. It is defined as the total income derived from either: (i) crop-only activity; (ii) livestock-only activity; (iii) mixed farming (crop and livestock); or (iv) off-farm activities. Off-farm activities include sources of livelihood other than crop and/or livestock, such as military service, government and private jobs, foreign remittances,....

The crop-only system (*Crop)* is mainly based on the availability of agricultural land and other input variables. The land constraint can be alleviated through the use of leases. An alternative specialized farming system is livestock-only system (*Livest*). This activity is based on the availability of strategic resources, mainly herd size. While a very small number of households are engaged only in crop farming (5%) or only in livestock farming (7%), more than half of the households (53%) in our sample are engaged in a mixed farming system, where both activities are practiced together (*CropLive*). In addition to the above farming activities, a third of the households in our sample (35%) are engaged in off-farm work, such as running their own non-agricultural business, serving in the government military or private sector,... (*Others*).

A second set of explanatory variables is related to land and capital used in agricultural activity. Regarding access to land, we have three variables that reflect land endowment. First, the total size of land owned and leased using tenancy contracts (*LandSize*). Second, land ownership (*LandOwn*) is a proxy for assessing property rights over the strategic asset of land [46]. Securing property rights over land is critical for smallholder households because even if the size of the plot is small (2 hectares on average) and thus economies of scale may be limited, at least an income can be secured. To overcome the land size constraint, the household may also have recourse to a tenancy agreement. We introduce a dummy variable (*ShareCrop*) if the household uses sharecropping to access additional land.

The other strategic asset is the fixed or variable capital used to ensure agricultural activity. Despite the fact that agricultural machinery can lead to higher productivity in cropping activities, poverty in this sector deprives a large number of smallholders from acquiring and using this equipment [47]. However, a small proportion of the surveyed households use some machinery (*FMachinery*) such as tractors, planters, threshers, harvesters, etc. The variable capital used in livestock is the livestock population. We use a measure of this population (*HerdSize*) that consists mainly of small and large ruminants, such as buffalo, cattle (cow), goat, and sheep. We expect all these variables to have a positive impact on net income by increasing the capital available for agricultural activity.

Considering the criticism of [42] about the neglect of socio-demographic variables in the study of farming systems, we introduce this set of variables in the regression. The first is the age (*AGE*) of the head of the household who manages the farm. Since an older smallholder is more skilled and thus better able to manage a farm, we expect this variable to increase total income [48]. Similarly, a higher level of education may improve the farmer’s productivity and ability to achieve optimal yields [22]. The number of years of schooling is used as a proxy for education (*EDU*). The average number of years of schooling is 2.5 and 42% of the respondents were illiterate. Another variable is the role of the family and its composition. In rural areas of Punjab, there is a general culture of living in a joint family system (*FmlySys*), where several generations live together. More than half of our respondents live under this type of family system.

We also construct a variable indicating the size of the family (*FmlySize*). In our sample, the average size is 5.37 members. We expect that a joint and larger family will increase total income, since less costly labor is available in such a family system. And also because, according to the imperfect labor supervision cost theory, family labor is more productive than hired labor due to better supervision [49]. In addition, in a joint family system, multiple generations work together and thus there is better supervision because older family members can better manage the work of younger members [50]. There is some multicollinearity between *FmlySize* and *FmlySys*, as well as between *LandOwn* and *LandSize*, but this does not generate a significant inflation of the standard errors. Therefore, we decided to include both pairs of variables in our regression.

To control for the spatial differences between the agro-climatic zones of the Province of Punjab, we introduce a set of regional variables. Indeed, under a vast and apparently homogeneous region, different contextual or sub-regional (district) realities may have some impact on the level of household income. [51] have confirmed that the apparent homogeneity of the vast irrigated plains in the Indo-Gangetic Plains (IGP) region masks significant diversity in rural assets, livelihood strategies, and outcomes. [40] provide a socio-economic analysis of five agro-climatic zones in Punjab, Pakistan. As explained above, our study focuses on only three zones: Rain-fed zone, mixed cropping zone, and cotton growing zone. We construct three dummies (*Rainfed*, *Mixcrop*, and *Cotwheat*) to indicate the location zone of the households. In Table 3 below, we report some socio-economic indicators of these different zones.

**Table 3:** Socio-economic characteristics of the three zones.

Indices	Mixed-cropping	Cotton-wheat	Rain-fed
Family Size	7.80	8.00	6.90
Illiteracy (%)	47.80	54.80	31.4
Urban pop (%)	26.88	20.76	28.60
Without land ownership (%)	52.40	57.50	50.00
Dependency Ratio	0.94	0.99	0.79
employees in rural labor force (%)	54.40	58.90	31.80

Source: [40].

Agriculture is highly dependent on rainfall and the land is rather flat in the rainfed zone. In this zone the dependency ratio on agriculture, defined as the ratio of the number of unemployed persons to the number of employed persons in farming household, is the lowest [52]. Due to job opportunities in the armed forces and government administration, members of the household have the access to off-farm activities. This is also the region with the lowest illiteracy rate, family size, and percentage of rural employment. This can be partly explained by the fact that it is also the zone with the highest urban population. On the other hand, the cotton-wheat region is dominated by agriculture and rural employment (around 60% of the working population and a dependency ratio close to 1) and a lower social status (highest illiteracy and family size) since the legitimate social model becomes the urban nuclear model with access to education. Between these two polar zones lies the mixed cropping zone.

In addition to these regional dummies, we also construct a proxy to capture the proximity of the village to an urban area (*Proximity*). For the region variables, we expect that households in the mixed cropping zone and the rainfed zones will have higher incomes compared to the cotton-wheat zone. Similarly, we expect that the closer the household’s village is to an urban area, the higher its income. The household may be able to get a higher price for its agricultural products or have access to better jobs.

Using these variables, Table 4 below shows the estimates of the linear regression model using OLS method. The estimates are robust to heteroskedasticity bias (Hubert-White sandwich estimator) and we also control for the possible endogeneity of the three activity dummies (*Crop*, *Livest*, *CropLive*) using an augmented regression test (Durbin-Wu-Hausman test).

**Table 4:** OLS estimates.

Variables	Coeff.	Standard errors
Crop	-0.056	0.124
Livest	-0.469 ***	0.168
CropLive	0.791 ***	0.207
AGE	0.065 **	0.030
EDU	0.075 ***	0.018
FmlySize	0.005	0.020
FmlySys	0.016	0.056
Landown	0.052	0.086
LandSize	0.079 ***	0.009
ShareCrop	0.018	0.077
FMachinery	0.065 *	0.038
HerdSize	0.248 ***	0.056
Cotwheat	Ref.	Ref.
Rainfed	0.001	0.097
Mixcrop	0.523 ***	0.719
Proximity	0.091 ***	0.025
Constant	10.540 ***	0.199
N = 360		
*Complementarity test* $\theta^{CL}=\left[\theta_{11}+\theta_{00}\right]-\left[\theta_{01}+\theta_{10}\right]\geq 0$	F(1, 346) = 10.15***	
Model	F(15, 344) = 24.18***	
R^2^	0.5120	

*Significant *at* 10%;

**Significant *at* 5%;

***Significant *at* 1%. Hubert-White sandwich estimator.

The results provide evidence of a clear complementary effect in the mixed farming system. First, the *CropLive* variable has a positive and significant impact on household income compared to the reference (*Others*).

Second, the complementarity test run confirms this result. This test indicates whether the adoption of a mixed farming activity generates a higher income compared to the specialized farming systems (only crop and only livestock activities). When we run the complementarity test, where the null hypothesis is the binding inequality in equation (7), we find clear evidence of a complementarity effect in mixed farming, as the null hypothesis is not rejected at 99%.

The OLS regression allows us to identify precisely the marginal effect of the choice of mixed farming, just by looking at the value of the estimated coefficient. Since we choose a log Cobb-Douglas specification for the farmers’ production function, the linear regression model of total income (6) has only linear terms. Therefore, OLS parameters and (average) marginal effects coincide [53,54]. Only in case of nonlinearity, e.g., quadratic effect of at least one explanatory variable, or in other statistical models such as logistic regression, which are nonlinear by design, the estimated coefficients generally differ from the (average) marginal effects [55].

The smallholder can then obtain on average an additional income of 791 Rupees (for an average total income of 12 010 Rupees) if he/she chooses mixed farming, all things being equal. This result of complementarity between crop and livestock activities within the farm provides a theoretical and empirical basis for the economic efficiency of mixed farming systems [12] and the additional income they generate for poor farmers [7,11,13].

Thirdly, with regard to the impact of the specialized farming systems, we obtain contrasting results. If the adoption of a crop-only activity (*Crop*) has a positive but nonsignificant effect on income, the livestock-only strategy (*Livest*) reduces the average level of income. The result suggests that livestock-only activity is primarily undertaken by the most impoverished smallholders. Nevertheless, this does not necessarily imply that livestock activity compromises the well-being of the smallholder.Livestock mainly provides an insurance and consumption smoothing mechanism by reducing income variability [31]. This can be explained by the possibility for the household to: (i) sell or consume part of the meat and milk production; (ii) sell the asset to buy other products from the markets, for example, in case of climatic or production shocks leading to low income [37]. Therefore, a risk-averse smallholder may have an interest in choosing a livestock production system even if it does not necessarily increase his income in the long run. This confirms the role of livestock activity in mixed farming as a risk mitigation and loss buffering device [14,28]. This is mainly through consumption smoothing, as shown by [31,33].

The non-human factors of production have the expected effect on income. First, larger land size increases household income. However, the specific type of land tenure, either ownership or sharecropping, has no significant effect. This is in contradiction with [46], for whom land tenure is an efficient system to ensure property rights over land and thus increase farmers’ income. Physical capital also seems to be a driver of income, as herd size and access to machinery variables (*Fmachinery*, *Herdsize*) are significant. This result suggests that what increases income in the livestock system is not the system itself, since we have shown above that the coefficient associated with this variable is negative. The crucial variable is the herd size, which is consistent with the result on the insurance and consumption smoothing function as shown by [31–33].

Regarding the human factor, the age of the farmer and his/her level of education increase the income by improving his/her productivity and ability to manage the activities of the farm. This is consistent with the predictions of [22,48]. In contrast, a joint family or a larger family has no effect on income, which is in contrast to [49,50].

Similarly, the regional variables have a mixed effect. While the coefficient associated with the mixed-crop zone is positive and significant, the coefficient associated with the rainfed zone is not significant. This can be explained by the fact that what matters in the rainfed zone is the characteristic of the urbanized area. And this is better captured by the *Proximity* variable, which has a positive and significant effect on income, confirming the prediction of [52].

There are three limitations of our study. First, although we were able to control for endogeneity bias by performing a Hu-Durbin-Watson test (see lines 356–357), it would be interesting to go further into the more general analysis of the potential endogeneity bias that unobserved heterogeneity can generate in activity decision and thus on the farmer’s income. A structural empirical model can be estimated to see if the complementarity between crop-livestock activities is robust to unobserved heterogeneity in Pakistan Punjab data. Second, even if recent studies suggest that mixed farming is still a major source of farm income in Punjab province even with the development of more specialized dairy farming [56], it would be interesting to see how the choice of agricultural activities, their complementarity, and their impact on the income of rural smallholders have evolved since 2014. A request for access to the latest Survey on rural smallholder farmers carried out by the Punjab government is underway. Finally, in addition to increasing smallholders’ income, management of mixed farming systems by women when they are the household’s head may also improve their social status and welfare. It will be interesting to explore this gender issue in future research.

## Conclusion and policy recommendations

Previous literature has mainly highlighted the synergistic effects between crop and livestock activities in mixed farming from an agronomic and environmental point of view. Some studies have also shown that mixed farming can provide economic benefits to reduce poverty among smallholder farmers in developing countries.

Our main contribution in this paper is to reconcile these two results and to show that synergies between crop and livestock activities can generate an (economic) complementarity effect that increases smallholders’ incomes more than the specialized activities (crop alone or livestock alone) and thus reduces poverty. To test for such an effect, we used a database of 360 farming households in Punjab, Pakistan.

Our main estimation result shows a significant and positive complementarity effect between crop and livestock activities that increases smallholders’ income whatever the size of the farm, while choosing to specialize (either crop or livestock) has no significant impact on income. This implies that smallholders may choose a mixed farming system not only because of its agronomic and environmental advantages, but also because it provides them with higher income than more specialized farming systems. Moreover, it is possible to measure the complementary effect directly. Our estimates show that, all things being equal, the smallholder can obtain an average additional income of 791 rupees (out of an average total income of 12,010 rupees) if he/she chooses mixed farming. Apart from the choice of activity, our estimation results show that the other variables that significantly increase the income of smallholders are the age and education level of the head of the household, as well as the size of the land, the size of the herd and access to urban markets. Our results also show that the positive impact of land expansion does not depend on the property rights regime. The additional land can be owned or rented (sharecropping). Therefore, a specific public policy aimed at increasing the income of smallholder farmers must prioritize the improvement of these key drivers, as a matter of priority.

## Supporting information

S1 Data XXXX.
(XLSX)
